# Cloning of a Novel 6-Chloronicotinic Acid Chlorohydrolase from the Newly Isolated 6-Chloronicotinic Acid Mineralizing Bradyrhizobiaceae Strain SG-6C

**DOI:** 10.1371/journal.pone.0051162

**Published:** 2012-11-30

**Authors:** Madhura Shettigar, Stephen Pearce, Rinku Pandey, Fazlurrahman Khan, Susan J. Dorrian, Sahil Balotra, Robyn J. Russell, John G. Oakeshott, Gunjan Pandey

**Affiliations:** 1 CSIRO Ecosystem Sciences, Australian Capital Territory, Australia; 2 Institute of Microbial Technology, Chandigarh, India; Université Paris-Sud, France

## Abstract

A 6-chloronicotinic acid mineralizing bacterium was isolated from enrichment cultures originating from imidacloprid-contaminated soil samples. This Bradyrhizobiaceae, designated strain SG-6C, hydrolytically dechlorinated 6-chloronicotinic acid to 6-hydroxynicotinic acid, which was then further metabolised via the nicotinic acid pathway. This metabolic pathway was confirmed by growth and resting cell assays using HPLC and LC-MS studies. A candidate for the gene encoding the initial dechlorination step, named *cch2* (for 6-chloronicotinic acid chlorohydrolase), was identified using genome sequencing and its function was confirmed using resting cell assays on *E. coli* heterologously expressing this gene. The 464 amino acid enzyme was found to be a member of the metal dependent hydrolase superfamily with similarities to the TRZ/ATZ family of chlorohydrolases. We also provide evidence that *cch2* was mobilized into this bacterium by an Integrative and Conjugative Element (ICE) that feeds 6-hydroxynicotinic acid into the existing nicotinic acid mineralization pathway.

## Introduction

Over the last two decades, neonicotinoids have risen to become one of the most widely used classes of insecticides against a broad spectrum of crop and domestic pests [1], [2]. In 2008, they accounted for around 24% of the total global insecticide market [2]. Neonicotinoids act selectively on the insect central nervous system as agonists of the nicotinic acetylcholine receptor (nAChR) and cause death by blocking the nicotinergic neuronal pathways [3]–[6].

**Figure 1:**
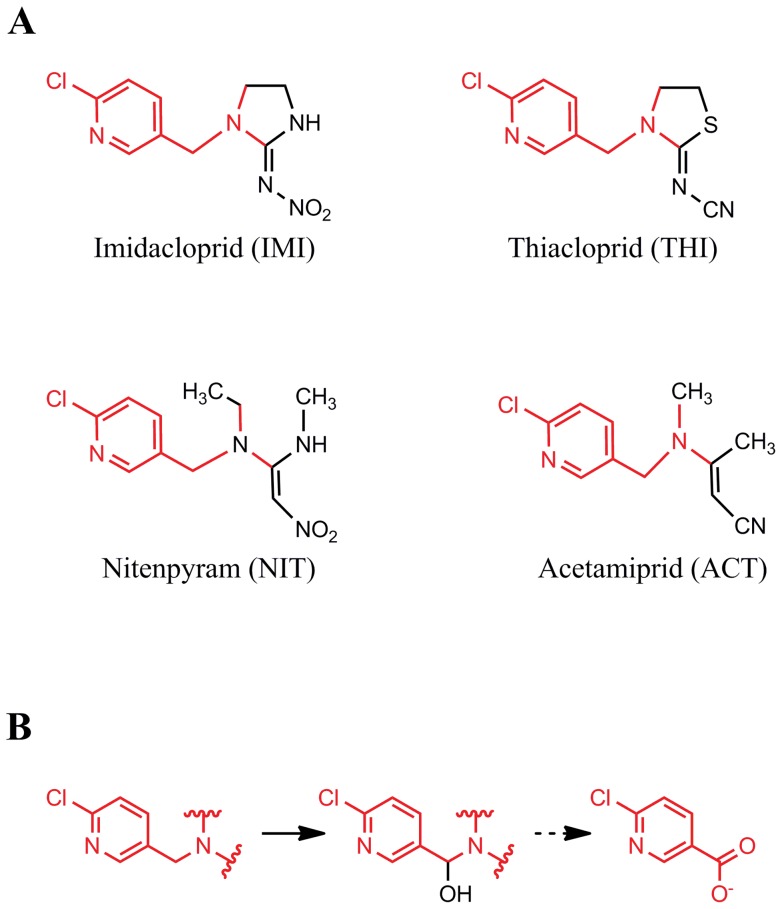
Chloropyridinylmethyl (CPM) neonitotinoids. Four commercial chloropyridinylmethyl (CPM) neonitotinoids (A) and their degradation to 6-chloronicotinic acid via methylene hydroxylation (B).

The seven major commercial neonicotinoids can be categorised as chloropyridinyls, chlorothiazolyls and tetrahydrofuryls on the basis of their N-heterocyclylmethyl moieties [7], [8]. However, there is also significant heterogeneity within the major chloropyridinyl category, with the chloropyridinylmethyl (CPM) group coupled with either a cyclic N-nitroimine moiety in imidacloprid (IMI), an N-cyanoimine moiety in thiacloprid (THI), an acyclic N-cyanoimine moiety in acetamiprid (ACT), or a 2-nitromethylene moiety in nitenpyram (NIT) (Figure 1) [7], [8].

The metabolism of commercial neonicotinoids has been extensively studied both in the environment and in various biological systems, in part because of increasing concerns about the toxicities of some metabolites [3], [4], [9]–[11]. One common early step for degradation of CPM neonicotinoids *in vivo* and in the environment involves N-methylene hydroxylation to an intermediate that spontaneously converts to 6-chloronicotinaldehyde, most of which is oxidized to 6-chloronicotinoic acid (6-CNA) [7]. To the best of our understanding there is no other source of 6-CNA in the environment. Although studies on the fate of 6-CNA in mice and spinach have established that it is removed from the system through various conjugated metabolites, there are no reports on the fate of 6-CNA in the environment [7]. 6-CNA has been found to accumulate as a major metabolite (0.5 to 1 ppm) in soils after 2 months of IMI application (7.2 ppm) [12].

This study was conducted with the aim of elucidating the environmental fate of 6-CNA. Here, we report isolation and characterization of a 6-CNA degrading soil bacterium and cloning of a novel 6-CNA dechlorinating gene-enzyme system from this bacterium. Furthermore, we provide biochemical and genomic evidence that 6-CNA is degraded via a pre-existing nicotinic acid (NA) catabolic pathway in this bacterium and that the gene encoding the 6-CNA dechlorinating gene-enzyme system has been acquired through horizontal gene transfer of an Integrative and Conjugating Element (ICE) [13].

## Materials and Methods

### Chemicals

Nicotinic acid (NA), 6-chloropyridine-3-carboxylic acid (6-CNA) and 6-hydroxypyridine-3-carboxylic acid (6-HNA) were purchased from Sigma-Aldrich Pty. Ltd, Australia. All other reagents were purchased from local vendors.

**Figure 2:**
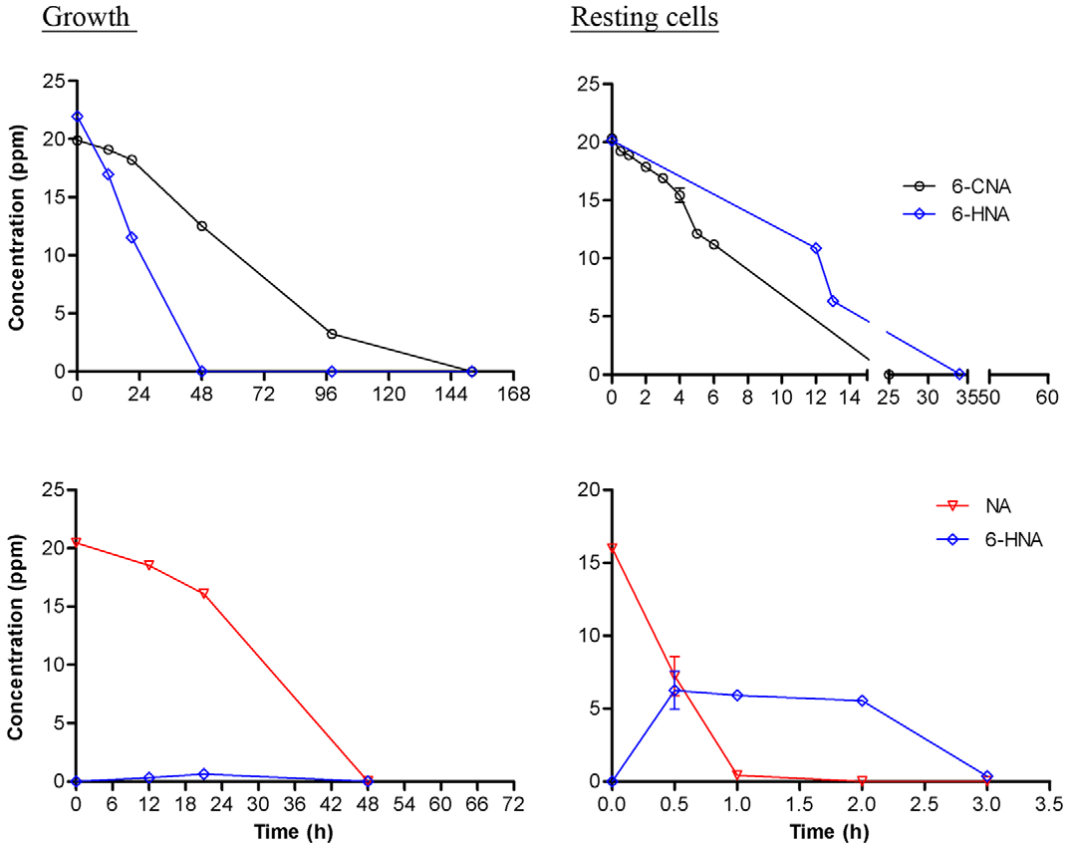
Growth and resting cells assays of SG-6C. Degradation kinetics of NA, 6-CNA, and 6-HNA as sole carbon sources in growth and resting cell studies by strain SG-6C. Values are the means of three replicates with standard deviations, if visible.

### Media and growth conditions

Mineral salt medium (MSM) used in this study was as described previously [14]. Quarter strength Luria Bertani medium with glycerol [QSLB; 2.5 g tryptone, 1.25 g yeast extract, 2.5 g NaCl, 20 g sodium succinate and 20 ml glycerol per litre] was used for growth of strain SG-6C at 30°C.

**Figure 3:**
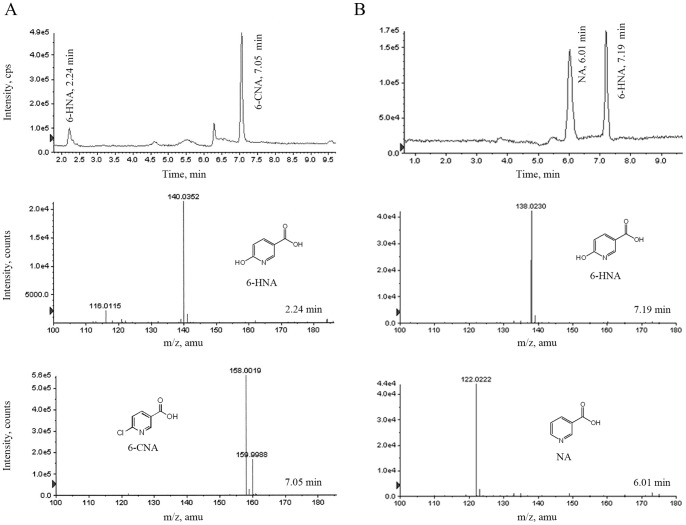
Appearance of 6-HNA as a metabolic intermediate of 6-CNA and NA in resting cells of strain SG-6C. A) LC-MS TOF Total Ion Chromatogram (TIC) showing appearance of 6-HNA in the supernatant of resting cells cultures supplemented with 6-CNA. Mass spectra of the two compounds are shown underneath the TIC. B) LC-MS TOF TIC showing appearance of 6-HNA in the supernatant of resting cells cultures supplemented with NA. Mass spectra the two compounds are shown underneath the TIC.

### Isolation of a 6-CNA degrading bacterial strain

Imidacloprid-exposed soil samples were collected from the Murrumbidgee Country Club, Australian National Territory (ACT), Australia. A permit to collect soil samples was obtained from Cambell Griggs on behalf of the club. MSM (50 ml) containing 50 ppm (0.3 mM) 6-CNA was inoculated with 1 g of pooled soil samples and incubated at 30°C for one week. This culture (3% v/v) was then transferred to fresh MSM (with 50 ppm 6-CNA) and then incubated at 28°C for another week and tested for 6-CNA degradation as described below. After 42 rounds of such enrichments, the growth medium was plated onto the QSLB plates and incubated for 1 week. Morphologically distinct individual colonies were then tested for 6-CNA degradation in liquid culture by LC-MS methods described below. The initial biochemical characterization of one strain found to mineralize 6-CNA, named strain SG-6C, was carried out by DSMZ, Germany.

**Figure 4:**
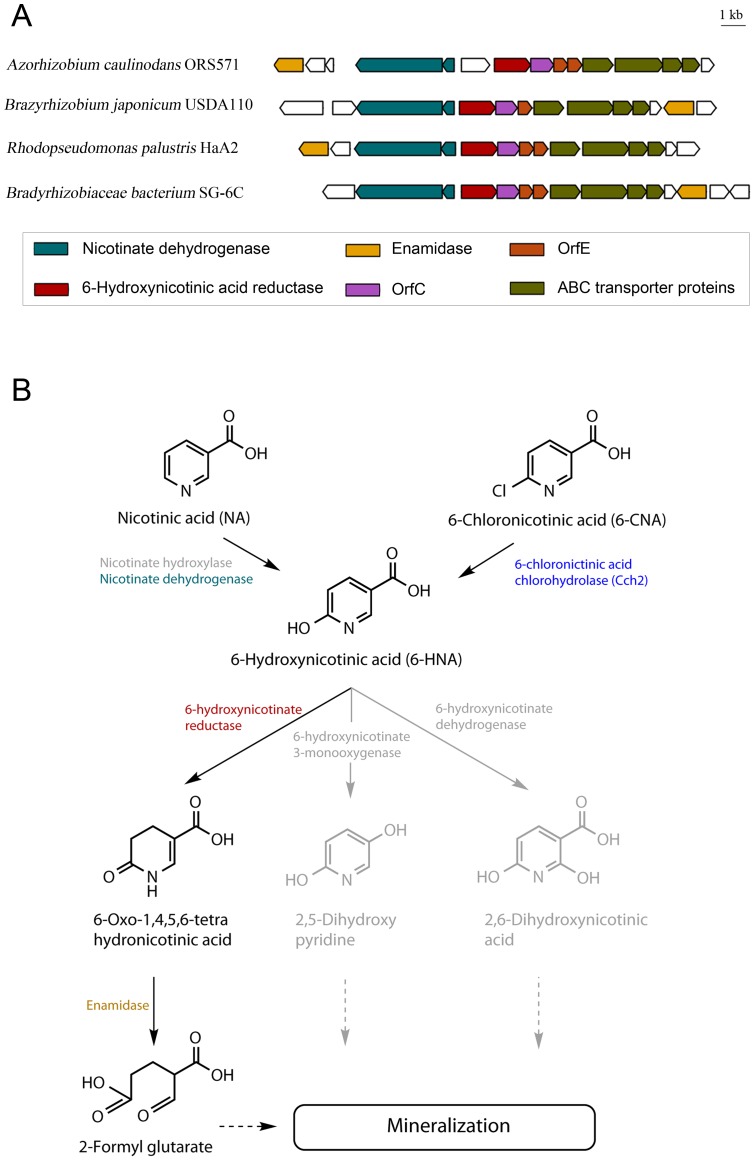
Nicotinic acid and 6-chloronicotinic acid degradation in SG-6C. A) Nicotinate degradation cluster in *Azorhizobium caulinodans* and selected Bradyrhizobiaceae strains. Genes were identified by Blast searches using the nicotinate degradation cluster of *Eubacterium barkeri* [23]. Genome accession numbers and locus tags for the displayed genes are as follows: *Azorhizobium caulinodans* ORS571 – NC_009937 (AZC_2804 to AZC_2790), *Bradyrhizobium japonicum* USDA110 – NC004463 (blr3818 to blr3830), *Rhodopseudomonas palustris* HaA2 – NC007778 (RPB_1671 to RPB_1658), Bradyrhizobiaceae bacterium SG-6C – AFOF01000023 (CSIRO_2076 to CSIRO_2090). Genes not conserved in the nicotinate degradation cluster are shown in white. B) Pathway for nicotinate and 6-chloronicotinate degradation in SG-6C. Nicotinate is metabolised via THON by the nicotinate degradation cluster shown above. 6-CNA is also metabolised via THON, after being converted to 6-hydroxynicotinate by Cch2. Pathways for nicotinate degradation in other organisms (not observed in strain SG-6C) are shown in grey.

### Characterization of a 6-CNA chlorohydrolase from strain SG-6C

Based on significant identities with metal-dependent chlorohydrolases, two open reading frames (ORFs; annotated as an amidohydrolase and a methyladenosine deaminase; accession numbers ZP_08626958.1 and ZP_08626964.1, respectively) from the genome sequence of strain SG-6C (Accession No. AFOF01000000; [15]) were selected and named as *cch1, cch2* (for 6-chloronicotinic acid chlorohydrolase). Both of these ORFs were cloned into pDEST17 (Invitrogen, CA), heterologously expressed in *E. coli* BL21-AI^TM^ (Invitrogen) cells and tested by the resting cells assay described above.

**Figure 5:**
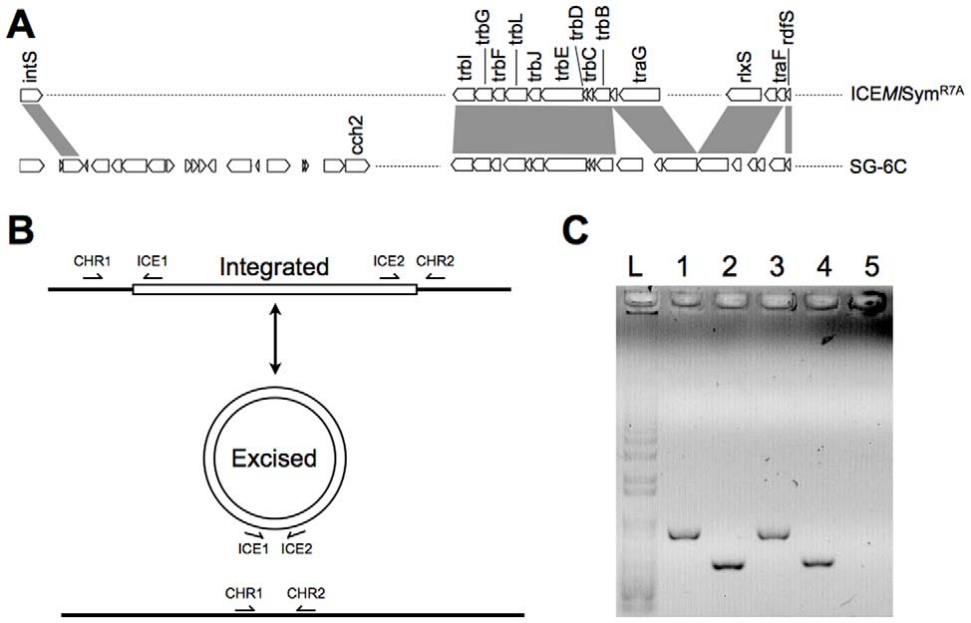
Identifying an ICE containing *cch2*. (A) Comparison of the *Mesorhizobium loti* symbiosis island (ICE*Ml*Sym^R7A^) and SG-6C. Blast matches for genes involved in the excision, conjugative transfer and integration of the symbiosis island are all found in SG-6C (indicated by grey shading). The location of *cch2* in the accessory gene region of the SG-6C ICE is indicated; (B) The two potential forms of the SG-6C ICE, integrated and excised. Binding sites of primers used to confirm the presence of both forms are indicated by half arrows; (C) PCR of strain SG-6C genomic DNA using primer combinations as indicated. L: 1kb+ ladder, 1: CHR1 & ICE1, 2: ICE2 & CHR2, 3: CHR1 & CHR2, 4: ICE1 & ICE2, 5: negative control.

For cloning into pDEST expression vectors, both the genes were PCR amplified using primers described in Table S1. The amplicons were cloned in pDONOR201 (Invitrogen) and transferred to pDEST17 using BP and LR reactions, respectively, following the manufacturer's instructions. The host line was *E. coli* BL-21-AI^TM^ (Invitrogen).

### Identification of an ICE

The genome sequence of SG-6C was used to design primers that amplified across the predicted integration site of an ICE (Table S1). PCRs were performed using prepared genomic DNA (QIAGEN) and Phusion High-Fidelity DNA Polymerase (Thermo Scientific, Australia). The PCR products were purified from a 1% agarose gel using a Gel Extraction Kit (QIAGEN) and sent to Micromon (Victoria, Australia) for sequencing.

### Elucidation of the 6-CNA catabolic pathway

Both resting cells and growth experiments were used to investigate the 6-CNA catabolic pathway in SG-6C.

For the resting cells experiments, a seed culture of strain SG-6C was prepared by growing the strain in QSLB at 30°C with shaking (180 rpm) for 48 h. The seed culture (6% v/v) was then inoculated into 1.6 L of QSLB and grown with shaking at 30°C for 24 h (OD_600_ ∼1.3–1.4). It was then centrifuged at 8000 g for 10 min at room temperature, and the pellet resuspended in 600 ml MSM and divided into five aliquots which were subjected to a second centrifugation at 8000 g for 10 min. Each pellet was then resuspended in 80 ml MSM containing 20 ppm of the compound of interest (NA, 6-CNA, or 6-HNA).

For the growth experiments, a seed culture prepared as above was inoculated (1% v/v) into 80 ml of MSM containing 20 ppm of the compound of interest.

Resting cells studies of *E. coli* cells containing pDEST17:CCH2 were performed in the same way as above except that the culture was grown in LB medium with 100 µg ml^−1^ ampicilin and induced with 0.2% arabinose.

Uninnoculated MSM containing substrate was used as a negative control in all assays.

In all experiments, 1 ml samples were collected at different time points and immediately filtered through 0.22 µM Miller GV Durapore 13 mm filters (Millipore, USA) and stored at 4°C until analysis by LC-mass spectrometry (LC-MS).

An Agilent 1100 series LC-MSD TOF (Agilent Technology, CA) was used for qualitative and quantitative analysis of all the substrates and metabolites. 6-CNA and 6-HNA were separated at 40°C on a Phenomenex column [Luna, 3µ, C18(2), 100A, 75×4.60 mm] using a gradient method consisting of water (A) and acetonitrile (B) (both containing 0.1 formic acid) as the mobile phase with a constant flow rate of 0.8 ml. min^−1^. The 12 min elution program was: (1) isocratic for the first 3.5 min (5% B); (2) gradually increased to 40% B by 4.0 min and maintained until 8.5 min; and (3) gradually decreased to 5% B by 9.0 min and maintained until 12 min. NA was separated at 25°C on an Obelisc N column (5 µ, 100 A, 4.6×250 mm; SIELC Technologies, IL) using water containing 100 mM ammonium formate (pH 4.5) and acetonitrile (20∶80% v/v) as a mobile phase at a flow rate of 1 ml. min^−1^. All reactants and products were monitored at 270 nm. The LC-MS TOF mass-spectrometer (Agilent Technology) electrospray ionization conditions were as described previously [16], except for NA analysis, which was performed in negative ionization mode.

## Results and Discussion

### Enrichment, isolation and characterization of SG-6C

A 6-CNA degrading enrichment culture was readily established from imidacloprid-exposed soil samples. The culture was first tested after the second round of enrichment and found to completely degrade 6-CNA. After 42 rounds of subculturing, the initial 50 ppm (0.6 mM) of 6-CNA in this enrichment culture was completely degraded within seven days. When serial dilutions of this enrichment culture were plated onto QSLB plates, three different morphologically distinct bacterial colonies appeared after seven days of incubation at 28°C. These colonies were picked, purified and individually tested for their 6-CNA mineralization capabilities in liquid MSM with 6-CNA as a sole carbon source. Only one bacterial colony showed complete degradation of 6-CNA (in 152 h; Figure 2) and this bacterium was designated as SG-6C.

Cells of strain SG-6C were found to be Gram-negative, motile, creamish-white rods of approximate size 2.5 µm that grew on MSM and nutrient agar but not on MacConkey agar, and were catalase-positive. The 16S rDNA gene of this bacterium (Accession No. GU324241) has 99% identity with the 16S rDNA genes of members of the genera *Oligotropha* (Accession No. AB099659), *Bradyrhizobium* (Accession No. AF208514), *Rhodopseudomonas* (Accession No. AB250616) and *Afipia* (Accession No. AF338177) in the family *Bradyrhizobiaceae*, making it difficult to assign a genus to strain SG-6C. Given these affinities, we have not assigned SG-6C to a genus but simply denote it as Bradyrhizobiaceae strain SG-6C. Detailed phylogenetic analysis of strain SG-6C is currently underway.

### 6-CNA catabolic pathway

In growth experiments containing 20 ppm of 6-CNA (0.1 mM) as the sole carbon source, a 1% v/v seed culture of strain SG-6C completely degraded the 6-CNA within 152 h (Figure 2). Resting cell studies with 6-CNA as carbon source were then performed to identify the initial steps in the 6-CNA catabolic pathway. A small peak (2.24 min retention time) that appeared only after six hours incubation was observed by LC-MS. This peak was identified as 6-hydroxynicotinic acid (6-HNA) by comparison of its LC retention time and mass profile with those of an authentic standard (Figure 3). 6-HNA results from the hydrolytic dechlorination of 6-CNA.

To determine if 6-HNA was a metabolite of the 6-CNA mineralization pathway, growth and resting cells experiments were therefore performed as above using 6-HNA as sole carbon source in place of 6-CNA. Strain SG-6C cells completely degraded 6-HNA in 48 and 34 h in growth and resting cells studies, respectively (Figure 2). No metabolite(s) were detected in LC-MS assays during either of these studies. The transient appearance of 6-HNA in the resting cells assay of 6-CNA and the mineralization of 6-HNA in growth and resting cells assays confirmed 6-HNA as a metabolic intermediate of the 6-CNA catabolic pathway in strain SG-6C.

6-HNA is the first metabolic intermediate in the three known nicotinic acid (niacin, vitamin B3; NA) catabolic pathways in bacteria [17]. To determine whether the 6-HNA resulting from hydrolytic dechlorination of 6-CNA is metabolised via the NA catabolic pathway in strain SG-6C, growth and resting cells studies were carried out to establish whether it can mineralize NA as a sole source of carbon. Cells of strain SG-6C completely degraded NA within 48 h and 1 h in growth and resting cell studies, respectively (Figure 2). As anticipated, 6-HNA transiently appeared with maximum concentrations of 0.6 and 6 ppm at 21 h and 30 min in the culture supernatants of growth and resting cells, respectively (Figures 2 and 3). The retention time and mass profile match of this peak with those of authentic 6-HNA confirmed that 6-CNA is hydrolytically cleaved to 6-HNA which is further degraded by the NA pathway in strain SG-6C.

As noted above, three different catabolic routes for the degradation of 6-HNA have been described in bacteria. These initiate with either: (1) decarboxylation to 2,5-dihydroxypyridine [18], [19]; (2) a second hydroxylation at a second position to yield 2,6-dihydroxynicotinate [20], [21]; and (3) reduction to 1,4,5,6-tetrahydro-6-oxonicotinate (THON) [22], [23]. The first two of these are reported in aerobic microorganisms such as various *Pseudomonas* sp. strains and *Bacillus niacini*, respectively. The third was discovered in the anaerobe *Eubacterium barkeri*, and has subsequently been reported in the aerobic *Azorhizobium caulinodans* [24]. BLASTP searches of the SG-6C Whole Genome Shotgun sequence (Accession No. AFOF01000000) for proteins known to be involved in nicotinate catabolism identified a homolog of the *E. barkeri* 6-hydroxynicotinate reductase (42% identity), suggesting that reduction via THON could form part of the NA degradation pathway. This 6-hydroxynicotinate reductase (Accession no ZP_08628994.1) is located in a gene cluster which is conserved across *A. caulinodans* ORS571 and other putative nicotinate catabolising proteobacteria (Figure 4) [23]. This cluster contains the genes responsible for the first three steps of the nicotinic acid degradation pathway as well as a putative nicotinate ABC transporter and two conserved proteins of unknown function. The pathway for complete catabolism of nicotinate in *A. caulinodans* involves conversion of THON into TCA cycle intermediates via glutarate [25]. It is likely that SG-6C follows the same pathway for the catabolism of nicotinate (and therefore hydrolytically dechlorinated 6-CNA) as *A. caulinodans*.

### Cloning the 6-chloronicotinic acid chlorohydrolase gene

BLAST was used to identify potential homologs of three atrazine (a chlorinated N-heterocyclic herbicide) dechlorinases, AtzA (Accession No. AAC64663.1), TriA (Accession No. AAG41202.1) and TrzN (Accession No. AAL39016.1), which might be able to hydrolytically dechlorinate 6-CNA to 6-HNA in the genome of strain SG-6C. Two ORFs, annotated to encode an amidohydrolase (Accession No. ZP_08626958.1) and a methylthiadenosine deaminase (Accession No. ZP_08626964.1), showed 32%, 32%, 25% and 33%, 34%, 23% sequence identities with AtzA, TriA and TrzN, respectively. These ORFs (named as *cch1* and *cch2*) share 67% sequence identity and occur in the same genomic region of SG-6C, 7 kb from each other (locus tags CSIRO_0014 and CSIRO_0020 in contig00002; Accession No. AFOF01000001.1). Apart from each other, the closest matches for these proteins are uncharacterised amidohydrolases from *Paenibacillus dendritiformis* (Accession No. ZP_09678326.1, 39% identity) and *Clostridium botulinum* (Accession No. YP_001254683.1, 35–37% identity). Other annotated genes in the immediate region of *cch1* and *cch2* include ABC transporter components, transcriptional and translational regulators, a transposase and two integrases.

Both *cch1* and *cch2* were cloned into the *E. coli* expression vector pDEST17 and subjected to resting cell studies. The *cch2* clones converted 70% of 20 ppm 6-CNA to 6-HNA in 6 hours, as shown by their m/z values in TOF and retention times in LC (data not shown). No activity was observed for the *cch1* clones. Unsuccessful attempts were made to purify the expressed protein.

### Presence of c*ch2* in an ICE

Upstream (16 kb) of *cch2* lies a putative integrase (CSIRO_0003; Accession No. ZP_08626947.1) that is a homolog of the P4-type symbiosis island integrase of *Mesorhizobium loti* (Accession No. AAC24508.1, 33% identity). The symbiosis island of *M. loti* strain R7A is a chromosomally integrated 502 kb element containing genes responsible for symbiotic growth with *Lotus* species [26]. This symbiosis island, a type ICE, is capable of excision and conjugative transfer to other bacterial species [27]. ICEs are prevalent in bacteria, with over 50% of the sequenced genomes in some clades containing putative ICEs [28].

Strain SG-6C contains a 139 kb putative ICE with homologs of the *M. loti* conjugative transfer proteins and the integrase, recombination directionality factor and relaxase required for excision and transfer of the element [29]. PCR amplification using primers designed to cross the predicted boundaries of the element confirm the presence of both the integrated and excised forms (Figure 5). Alignments of amplicons identify a 48 bp sequence that is the predicted integration site of the element (Figure S1). It is common for ICEs to integrate in a tRNA sequence and a tRNAscan-SE search [30] of the amplified sequences identifies a tRNA^Glu^ in the integration site. To the best of our knowledge, this is the first ICE reported to integrate into a tRNA^Glu^ gene.

Apart from the core machinery, ICEs also contain variable regions that are known to be involved in numerous functions including symbiosis [26], pathogenicity [31] and antibiotic resistance [32]. The ICE in SG-6C does not contain any functional regions previously observed in ICEs, and most of the 138 ORFs within it have no predicted function (Table S2). However, the identification of this ICE in SG-6C provides the mechanism for the acquisition of 6-CNA degradation by the strain. Conjugation and integration of the ICE brought *cch2*, which converts 6-CNA into 6-HNA, into strain SG-6C. The 6-HNA is then mineralised by the pre-existing nicotinic acid degradation pathway in SG-6C.

The presence of *cch2* in an ICE suggests that it may occur in the genomes of other species that also have genes encoding the requisite downstream pathway for 6-CNA degradation. The 48 bp integration site is found in the genomes of a number of Bradyrhizobiaceae, including some putative NA degrading strains (eg. *Bradyrhizobium* sp. BTAi1 and *R. palustris* HaA2). However, the closest match of *cch2* in these genomes has a maximum identity of below 30%. It will be interesting to determine whether (near-) identical versions of *cch2* are found in other, yet to be discovered, 6-CNA and/or CPM neonicotinoid mineralizing bacteria, as has been found for the mobile genetic element-associated genes encoding the upstream components of the catabolic pathways for other synthetic chemical pesticides and herbicides released into the environment over the last 60 years (eg. AtzA, OpdA, LinA and LinB) [33]–[37].

## Supporting Information

Figure S1 **Integration site of the SG-6C ICE.** Section of the sequence alignment of the PCR amplicons from across the predicted boundaries of the SG-6C ICE. A 48 bp sequence (highlighted) is observed in all amplicons and is the predicted integration site of the element. Sequence names refer to the primer combinations used to perform the PCRs.
(DOCX)

Table S1 Primers used in this study.
(DOCX)

Table S2 ORFs on the SG-6C ICE. Details of the ORFs found on the SG-6C ICE. Annotated functions and size for each ORF are shown, as well as the percent identity to the best BLAST hit. An e-value cutoff of 0.1 was used to construct this list.
(XLSX)
