# Supplementary material for: Cloning of a Novel 6-Chloronicotinic Acid Chlorohydrolase from the Newly Isolated 6-Chloronicotinic Acid Mineralizing Bradyrhizobiaceae Strain SG-6C
Source: PLoS One. 2012 Nov 30;7(11):e51162. doi: 10.1371/journal.pone.0051162 (PMC3511419; doi:10.1371/journal.pone.0051162)
Supplement: Figure S1 — Integration site of the SG-6C ICE. Section of the sequence alignment of the PCR amplicons from across the predicted boundaries of the SG-6C ICE. A 48 bp sequence (highlighted) is observed in all amplicons and is the predicted integration site of the element. Sequence names refer to the primer combinations used to perform the PCRs. (DOCX) [file pone.0051162.s001.docx]

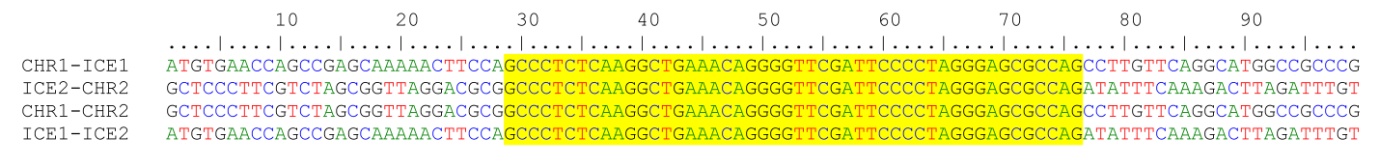


**Figure S1. Integration site of the SG-6C ICE.**

Section of the sequence alignment of the PCR amplicons from across the predicted boundaries of the SG-6C ICE. A 48 bp sequence (highlighted) is observed in all amplicons and is the predicted integration site of the element. Sequence names refer to the primer combinations used to perform the PCRs.
