# Supplementary material for: Cloning of a Novel 6-Chloronicotinic Acid Chlorohydrolase from the Newly Isolated 6-Chloronicotinic Acid Mineralizing Bradyrhizobiaceae Strain SG-6C
Source: PLoS One. 2012 Nov 30;7(11):e51162. doi: 10.1371/journal.pone.0051162 (PMC3511419; doi:10.1371/journal.pone.0051162)
Supplement: Table S1 — Primers used in this study. (DOCX) [file pone.0051162.s002.docx]

Table S1: Primers used in this study

| Primer Name | Sequence |
| --- | --- |
| CHR1 | GGGAATACAGTCCCCACACC |
| CHR2 | GCGGATATGAAGGGTCTGAA |
| ICE1 | ATCCGTCGAATAAGCAATGG |
| ICE2 | TGGTTCTCATCAACGCAGAG |
| CCH1-GatewayForward | GGGGACAAGTTTGTACAAAAAAGCAGGCTTAATGCATCTATTGGTGATT |
| CCH1-GatewayReverse | GGGGACCACTTTGTACAAGAAAGCTGGGTATCAAATCACCGGCCATAC |
| CCH2-GatewayForward | GGGGACAAGTTTGTACAAAAAAGCAGGCTTAATGAGCCTGATCGCAATTA |
| CCH2-GatewayReverse | GGGGACCACTTTGTACAAGAAAGCTGGGTATCAATGGACGGGCCACCG |
